# Impact of Carbon Felt Electrode Pretreatment on Anodic Biofilm Composition in Microbial Electrolysis Cells

**DOI:** 10.3390/bios11060170

**Published:** 2021-05-26

**Authors:** Sabine Spiess, Jiri Kucera, Hathaichanok Seelajaroen, Amaia Sasiain, Sophie Thallner, Klemens Kremser, David Novak, Georg M. Guebitz, Marianne Haberbauer

**Affiliations:** 1 K1-MET GmbH, Stahlstrasse 14, 4020 Linz, Austria; amaia.sasiain@k1-met.com (A.S.); sophie.thallner@k1-met.com (S.T.); marianne.haberbauer@k1-met.com (M.H.); 2 ACIB GmbH (Austrian Centre of Industrial Biotechnology), Krenngasse 37/2, 8010 Graz, Austria; guebitz@boku.ac.at; 3 Department of Biochemistry, Faculty of Science, Masaryk University, Kamenice 753/5, 62500 Brno, Czech Republic; jiri.kucera@sci.muni.cz (J.K.); dnovak@med.muni.cz (D.N.); 4 Linz Institute for Organic Solar Cells (LIOS), Institute of Physical Chemistry, Johannes Kepler University Linz, Altenberger Strasse 69, 4040 Linz, Austria; seelajaroen.h@gmail.com; 5 Department of Agrobiotechnology, Institute of Environmental Biotechnology, University of Natural Resources and Life Sciences Vienna, Konrad-Lorenz-Strasse 20, 3430 Tulln an der Donau, Austria; klemens.kremser@boku.ac.at

**Keywords:** bioelectrochemical system, bioelectrodes, biosensor, electrode pretreatment, metagenomic analysis, microbial communities

## Abstract

Sustainable technologies for energy production and storage are currently in great demand. Bioelectrochemical systems (BESs) offer promising solutions for both. Several attempts have been made to improve carbon felt electrode characteristics with various pretreatments in order to enhance performance. This study was motivated by gaps in current knowledge of the impact of pretreatments on the enrichment and microbial composition of bioelectrochemical systems. Therefore, electrodes were treated with poly(neutral red), chitosan, or isopropanol in a first step and then fixed in microbial electrolysis cells (MECs). Four MECs consisting of organic substance-degrading bioanodes and methane-producing biocathodes were set up and operated in batch mode by controlling the bioanode at 400 mV vs. Ag/AgCl (3M NaCl). After 1 month of operation, *Enterococcus* species were dominant microorganisms attached to all bioanodes and independent of electrode pretreatment. However, electrode pretreatments led to a decrease in microbial diversity and the enrichment of specific electroactive genera, according to the type of modification used. The MEC containing isopropanol-treated electrodes achieved the highest performance due to presence of both *Enterococcus* and *Geobacter*. The obtained results might help to select suitable electrode pretreatments and support growth conditions for desired electroactive microorganisms, whereby performance of BESs and related applications, such as BES-based biosensors, could be enhanced.

## 1. Introduction

Fossil fuels, such as coal and oil, are the primary sources of global energy supply. However, their combustion leads to greenhouse gas emissions, such as CO_2_, causing rising global temperatures and further climate changes [1]. Therefore, new sustainable technologies for energy production and renewable electricity storage are needed to tackle global warming and the energy crisis [2]. In the past decade, bioelectrochemical systems (BESs) have gained increasing interest, not only for sustainable energy generation and simultaneous wastewater treatment [3], but also for storage of renewable energy as high-value chemicals [4]. With this emerging technology, electricity can be converted into chemical energy and vice versa by employing electroactive microorganisms immobilized on BES electrodes, catalyzing the required redox reactions [5,6]. Electroactive microbes can either receive electrons from the electrode by substrate reduction [7] or pass electrons onto the electrode by substrate oxidation [3]. The first report describing electricity-producing microorganisms was published in 1911 by M.C. Potter [8]. Extracellular electron transfer can take place via direct electron transfer, mediated electron transfer using reusable intermediates, and indirect electron transfer, which is similar to mediated electron transfer but uses fermentative metabolites [9].

**Figure 1:**
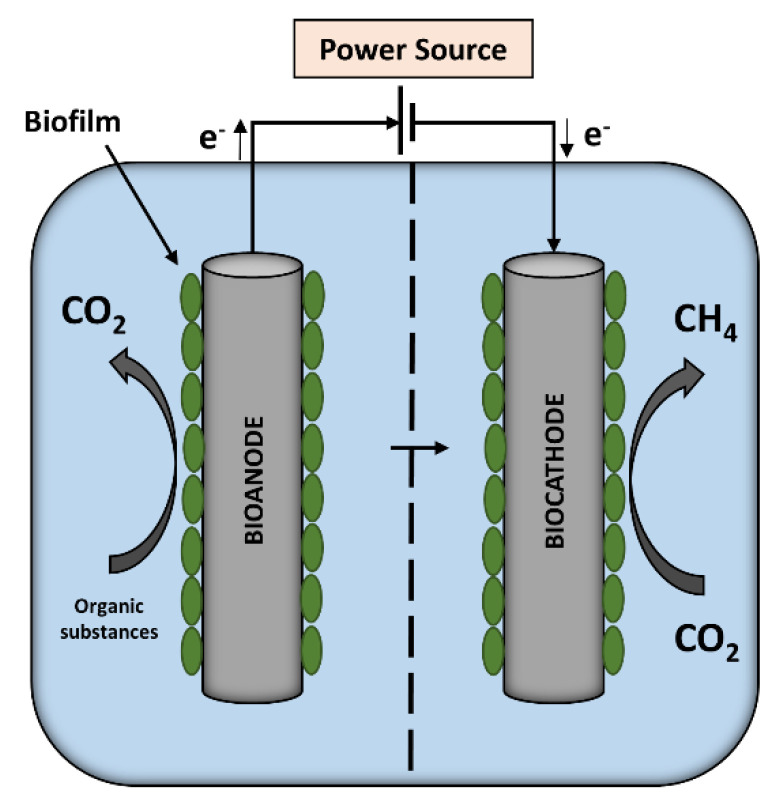
Microbial electrolysis cell (MEC) with an organic substance-oxidizing bioanode combined with a methane-producing biocathode.

Depending on electricity production or energy consumption, BESs can be classified as a microbial fuel cell (MFC) or as a microbial electrolysis cell (MEC) [5]. In recent years, researcher efforts regarding MFC technology expanded from wastewater treatment and electricity generation to other fields, such as bioremediation, desalination, and biosensors. In particular, the latter fields attracted great interest for detection of toxic chemicals and for monitoring the biological oxygen demand, due to their high sensitivity and applicability for remote sites because of their self-power ability [2]. However, there is still room for improvement for these MFC-based biosensors’ the electrode material [10] and the electroactive biofilm consortia, in particular, play key roles [11]. In MFCs, microbes are attached to bioanodes and catalyze the oxidation of organic substrate [12], whereas microbes in MECs are typically adhered to biocathodes [13], catalyzing reactions for the production of high-value molecules/chemicals, such as methane [7], formic acid [14], or ethanol [15]. In MECs, an external power supply is necessary to overcome thermodynamic barriers and fulfill the reduction reaction [16]. However, a bioanode can be combined with a biocathode (as shown in Figure 1). The bioanode supplies energy for the cathodic reduction, resulting in reduced external energy demand, for, e.g., methane production, unlike an abiotic anode [17,18,19,20]. This specific energy-efficient combination of bioelectrodes has attracted significant research interest to obtain cheaper and more sustainable cathodes [21] for denitrification process [22] and methane generation [23,24,25], similar to this study.

At the MEC bioanode, electrons are released by the oxidation of organic substrates, such as glucose (as described in Equation (1)), whereas electrons are consumed to reduce CO_2_ to methane at the MEC biocathode (Equation (2)).

C_6_H_12_O_6_ + 6H_2_O → 6CO_2_ + 24H^+^ + 24e^−^ (1)

CO_2_ + 8H^+^ + 8e^−^ → CH_4_ + 2H_2_O (2)

Carbon-based materials, such as carbon felt [26], carbon paper [27], or carbon mesh, are widely used as electrode materials for BESs, due to their biocompatibility, high surface area, chemical stability, and conductivity [28,29]. To further improve these properties, several attempts (such as physical and chemical treatments) have been made to modify their surfaces to enhance microbial attachment and increase the surface area for electron exchange between microorganisms and the electrode [30,31], as these are critical to the efficiency of BESs and related applications, such as MFC-based biosensors [10]. In particular, the modification with positively charged materials, such as ammonia or chitosan, has attracted great attention. The latter, an amino- and hydroxyl-group rich polysaccharide, is known as a commonly occurring biopolymer, with excellent properties regarding biocompatibility, hydrophilicity, film-forming ability, and chemical and mechanical stability [32,33], improving the adhesion and interaction of microbes and electrodes [34]. Furthermore, the phenazine dye, neutral red, is used in various applications (e.g., for medical purposes and as a pH indicator) [35], and in BESs, it is utilized as an efficient electron mediator [36]. Through electrochemical polymerization, neutral red can form a polymer on carbon-based materials, referred to as poly(neutral red), enhancing microbial electrosynthesis by improving electron transfer processes [37]. Moreover, carbon-based electrodes can be easily pretreated with isopropanol and hydrogen peroxide by simply reducing functional groups on the electrode surface [38].

Additionally, in our previous study, we focussed mainly on the enhanced performance parameters of MECs due to electrode pretreatments. However, little attention was paid to the impact of the pretreatment methods on bacterial community composition-enriched electrodes. Therefore, the effects of three different electrode pretreatment methods on anodic microbial consortium were investigated in this study. Metagenomic analysis was performed at untreated, poly(neutral red), chitosan, and isopropanol pretreated carbon felt bioanodes to observe and compare the enrichments of electrochemically active communities, with respect to the specific pretreatment method.

## 2. Materials and Methods

### 2.1. MEC Setup

Two-chamber H-cell type reactors, with a working volume of 220 mL per chamber, separated by a proton exchange membrane (Nafion 117, Chemours, Wilmington, DE, USA), were used for all experiments. The membrane was pretreated sequentially by boiling in 30% (*v*/*v*) H_2_O_2_, deionized (DI) water, 0.5M H_2_SO_4_, and DI water, according to a modified procedure [39]. Each step was carried out at 80 °C for 1 h. Carbon felt was selected as the electrode material (70 × 25 × 0.6 mm^3^, Alfa Aesar, Heysham, UK) due to its high surface area, which facilitates microbial adhesion, and for its high electrical conductivity [27]. As a reference electrode, an Ag/AgCl (3 M NaCl) electrode (ProSense, Oosterhout, The Netherlands) was inserted in the anode chamber. All voltages throughout this study are reported, with respect to Ag/AgCl (3 M NaCl; +209 mV vs. standard hydrogen electrode, SHE).

A phosphate buffer solution (PBS) was used as anolyte and catholyte for all MECs, consisting of the following components (per liter); 3 g KH_2_PO_4_, 2.5 g K_2_HPO_4_, 0.13 g NaCl, 0.31 g NH_4_Cl, 6 g NaHCO_3_, 0.04 g MgSO_4_∙7H_2_O, 12.5 mL trace element solution SL 10 (DSMZ 320), and 5 mL vitamin solution (DSMZ 141). The anode and cathode chambers of all MECs were inoculated with 20 mL of sewage sludge collected from a wastewater treatment plant in Upper Austria. Before inoculation, solid contaminants were removed from sewage sludge by centrifugation at 2150× *g* for 10 min. The chambers were then filled with 200 mL of PBS.

Different pretreatment methods for carbon felt electrodes were tested. Chitosan pretreatment was selected, as it coats the neutral charged carbon felt with a positively charged layer, promoting the interactions between Gram-negative cells and the electrode [34], as well as neutral red polymerisation, which changes the surface structure of carbon felt electrodes, caused by poly(neutral red) film formation, acting as redox mediator [35]. Meanwhile, isopropanol pretreatment reduces functional groups, leading to an increase in electron transfer properties and microbial adhesion on carbon felt electrodes [38]. Untreated electrodes were used as a control and designated MEC1. Electrodes from MEC2 and MEC3 were pretreated with poly(neutral red) and chitosan, respectively, as previously described [40]. In MEC4, carbon felt electrodes were pretreated, according to a modified procedure described by Cheng et al. [38]. The carbon felt electrode was washed with DI water and dried thoroughly at 100 °C. The electrode was then soaked in 30% (*v*/*v*) isopropanol overnight, rinsed with DI water, and dried. Afterwards, the carbon felt electrode was steeped in 30% (*v*/*v*) H_2_O_2_ for 24 h, washed with DI water, and dried at 100 °C for subsequent usage.

Anolyte and catholyte were continuously mixed at 70 rpm using a magnetic stirrer IKA RCT basic (Staufen, Germany). Electrochemical experiments were carried out via three electrode setups, using potentiostat PM-100 Jaissle Elektronik GmbH (Münster, Germany) and IVIUM CompactStat (Eindhoven, The Netherlands). The MECs were operated in batch mode at room temperature by applying a constant potential of 400 mV vs. Ag/AgCl (3 M NaCl) on the anode while monitoring the current flow.

### 2.2. MEC Operation

During the adaptation phase, carbon sources were supplied once a week into the anode chamber to simulate synthetic wastewater (per liter); 0.138 g peptone/trypsin, 0.075 g yeast extract, 0.088 g C_2_H_3_NaO_2_, and 0.37 g C_6_H_12_O_6_∙H_2_O. At each feeding, half of the anolyte was replaced with freshly prepared PBS, containing synthetic wastewater. The cathode chamber was fed with 1 g L^−1^ C_6_H_12_O_6_∙H_2_O only during the adaptation phase to promote microbial growth. The adaptation phase targeted microbial community formation, as well as the stabilization of bioelectrodes. Successfully developed MECs were indicated by producing an average of 2 mA. After adaptation for 4 weeks, 200 mL of the catholyte was replaced with fresh PBS. During batch experiments, MECs anodes were fed two times per week, replacing half of the anodic solutions with freshly prepared PBS containing synthetic wastewater, as described above (600 mg COD L^−1^), whereas the cathodes were supplied with pure CO_2_ as the only carbon source. In total, 8 feeding cycles were repeated with each MEC. Before each feeding, liquid and gas samples were taken from anodes and cathodes, respectively. Every 4 weeks, 200 mL of cathodic solution was replaced with fresh PBS to provide microorganisms with sufficient trace elements and vitamins and to stabilize the pH. MEC chambers were maintained under anaerobic conditions during the experiments.

### 2.3. Analytics and Calculations

A chemical oxygen demand (COD) test was used to measure the number of organic compounds in the samples. First, a 2 mL liquid sample was mixed with a reagent in Nanocolor COD 160 and 1500 test tubes (Macherey-Nagel, Düren, Germany) and then heated in the Nanocolor Vario Mini heating block (Macherey-Nagel, Düren, Germany) for 2 h at 148 °C. Subsequently, the tubes were removed from the heating block and allowed to cool down to room temperature and shaken after 10 min. The COD concentration was measured using the Compact Photometer PF-3 (Macherey-Nagel, Düren, Germany) at 620 nm. The COD removal efficiency was calculated using Equation (3), in which Δ*COD* represents the removed COD and *COD_IN_* is the COD of a provided substrate.

(3) $$COD\;removal\;efficiency\;\left(\%\right)=\left(\frac{\Delta COD}{COD_{IN}}\right)\times 100$$

The anodic coulombic efficiency (*CE*) was calculated using Equation (4), including the following terms: the molar mass of oxygen (*M* = 32 g mol^−1^), the recorded current integrated (*I*) over time (*t*), the number of electrons exchanged per mole of oxygen (four), the Faradaic constant (*F* = 96,485.33 C mol^−1^), the working volume of the anode chamber (*V* = 0.22 L), and the Δ*COD*.

(4) $$CE\;anode\;\left(\%\right)=\frac{M\times \int_{0}^{t}Idt}{4\times F\times V\times \Delta COD}\times 100$$

Before each feeding, a 2 mL gas sample was taken for methane analysis from cathodic headspace using a gastight syringe (Hamilton 1000, Reno, NV, USA). The sample was injected into the Thermo Scientific Gas Chromatograph Ultra (Thermo Fisher Scientific, Waltham, MA, USA), equipped with a thermal conductivity detector. The cathodic *CE* was calculated using Equation (5), encompassing the methane production in m^3^ (*V_CH_*_4_), the number of electrons needed for the reduction of CO_2_ to methane (eight), the Faradaic constant (*F*), the molar volume (*V_m_* = 0.0252 m^3^ mol^−1^), the recorded current (*I*), and the reaction time (*t*).

(5) $$CE\;cathode\left(\%\right)=\frac{V_{CH4}\times 8\times F}{Vm\times \int_{0}^{t}Idt}\times 100$$

For metagenomic analysis, biofilm was scraped from all MECs bioanodes after 1 month of batch experiments, resuspended in Tris-EDTA buffer, and then frozen at −80 °C. Further, the anolyte from MEC4 was removed and frozen at −80 °C. Sewage sludge inoculum was centrifuged at 2150× *g* for 10 min and the supernatant was frozen at −80 °C.

### 2.4. 16S rRNA Gene Amplicon Sequencing

Bacterial DNA was isolated using the DNeasy UltraClean Microbial Kit (Qiagen, Hilden, Germany), according to the manufacturer’s instructions. The V4 highly variable region was selected for amplification with unique barcoded primers (Supplementary Table S1). The primer sequences, consisting of an Illumina adapter (P5 or P7), an 8-nt index sequence representing the unique barcode, a 10-nt pad sequence, a 2-nt linker, and a specific sequence of the V4 region, were modified, according to Pichler et al. [41]. PCR amplification was performed using Platinum II Taq Hot-Start DNA Polymerase (Thermo Fisher Scientific, Waltham, MA, USA), as follows: initial DNA denaturation step at 94 °C for 3 min, 35 cycles of DNA denaturation at 94 °C for 45 s, annealing at 52 °C for 60 s with a 50% thermal ramp, extension at 72 °C for 90 s, and a final extension step at 72 °C for 10 min. The amplification product was purified by AMPure XP beads (Beckman Coulter, Brea, CA, USA), following the manufacturer’s instructions. The Qubit 4.0 fluorometer (Thermo Fisher Scientific, Waltham, MA, USA) and FragmentAnalyzer (Advanced Analytical Technologies, Inc., Santa Clara, CA, USA) were then used to determine the quality of the libraries. The library was sequenced using a MiniSeq System (Illumina, San Diego, CA, USA) with MiniSeq Mid Output Kit (300 cycles). Raw fastq reads were processed in R software (v4.0.3) using the open-source package DADA2 (v1.16.0) [42]. The DECIPHER package was used for multiple alignments with the phangorn package to build a phylogenetic tree, and the phyloseq package was used for subsequent phylogenetic analysis, as described earlier [43]. A summary of all amplicon sequence variants (ASVs) is shown in Supplementary Table S2. A fivefold quantitative difference in the representation of taxa was considered significant and evaluated as a qualitative change. Datasets generated and analyzed during the current study are available in the NCBI Sequence Read Archive under project number BioProject ID: PRJNA718863.

## 3. Results and Discussion

In order to investigate the influence on developing microbial populations, three different carbon felt electrode pretreatments were tested. MEC1 was equipped with untreated electrodes as the control, while the other MECs were equipped with poly(neutral red)- (MEC2), chitosan- (MEC3), and isopropanol-treated (MEC4) electrodes. Detailed information on the long-term performance of MEC1–3 has previously been reported [40]. This work obtains the results describing the performance parameters of MEC4. Table 1 provides an overview and compares performance parameters of MEC1–3 from our previous study [40] and MEC4 from this work after 1 month of batch operation.

During oxidation of organic substrates on the anode of MEC4, a COD removal rate of 69 ± 18 mg L^−1^ d^−1^ and a CE of 75 ± 12% was reached. Methane production in the cathode chamber of MEC4 was detected at a rate of 0.44 ± 0.12 mmol L^−1^ d^−1^, resulting in a cathodic CE of 58 ± 10%. Methane production rates of biocathodes are reported in a broad range between 0.13 to 30 L m^−2^, caused by a variety of system setups and different designs [44]. In comparison, methane production projected on cathode surfaces was 0.34 L m^−2^ for MEC1, 1.26 L m^−2^ for MEC2 and MEC3, and 1.35 L m^−2^ for MEC4, which fits in this bandwidth but leaves room for further improvements. The COD removal efficiency of MEC4 was, on average, 76 ± 22%, which is comparable to results from previous reports [23], in which a methane producing MEC was also operated by controlling the anode potential, but anodic CE in this work was 30% lower than the reported anode potential (105%).

Comparing MEC1–4 showed that pretreatment of carbon felt lead to considerably enhanced performance of bioelectrochemical cells. Positively charged chitosan coating may enhance interactions between the electrode and Gram-negative microorganisms [34], which was also evident for chitosan pretreated MEC3, producing a Q rate two times higher than the untreated MEC1. This corresponds to a previous study [34], in which positively charged chitosan coating improved the interaction between the electrode and the Gram-negative microorganism *Sporomusa ovata*. Further, the immobilization of mediators, such as neutral red, on carbon-based materials (MEC2) potentially increase current production. The highest Q rate (137 ± 36 C d^−1^) was observed at MEC4 with isopropanol pretreated electrodes. Isopropanol may reduce surface functional groups, such as nitrogen or oxygen [38]. The reduction of oxygen functional groups coupled to enhanced electrode performance has also been reported in earlier studies [45,46]. Further, methane production rates on all pretreated cathodes were considerably higher (0.41 to 0.44 mmol L^−1^ d^−1^) than the untreated electrode (0.11 mmol L^−1^ d^−1^), possibly caused by enhanced interactions between microbes and electrodes or boosted by higher Q rates.

**Figure 2:**
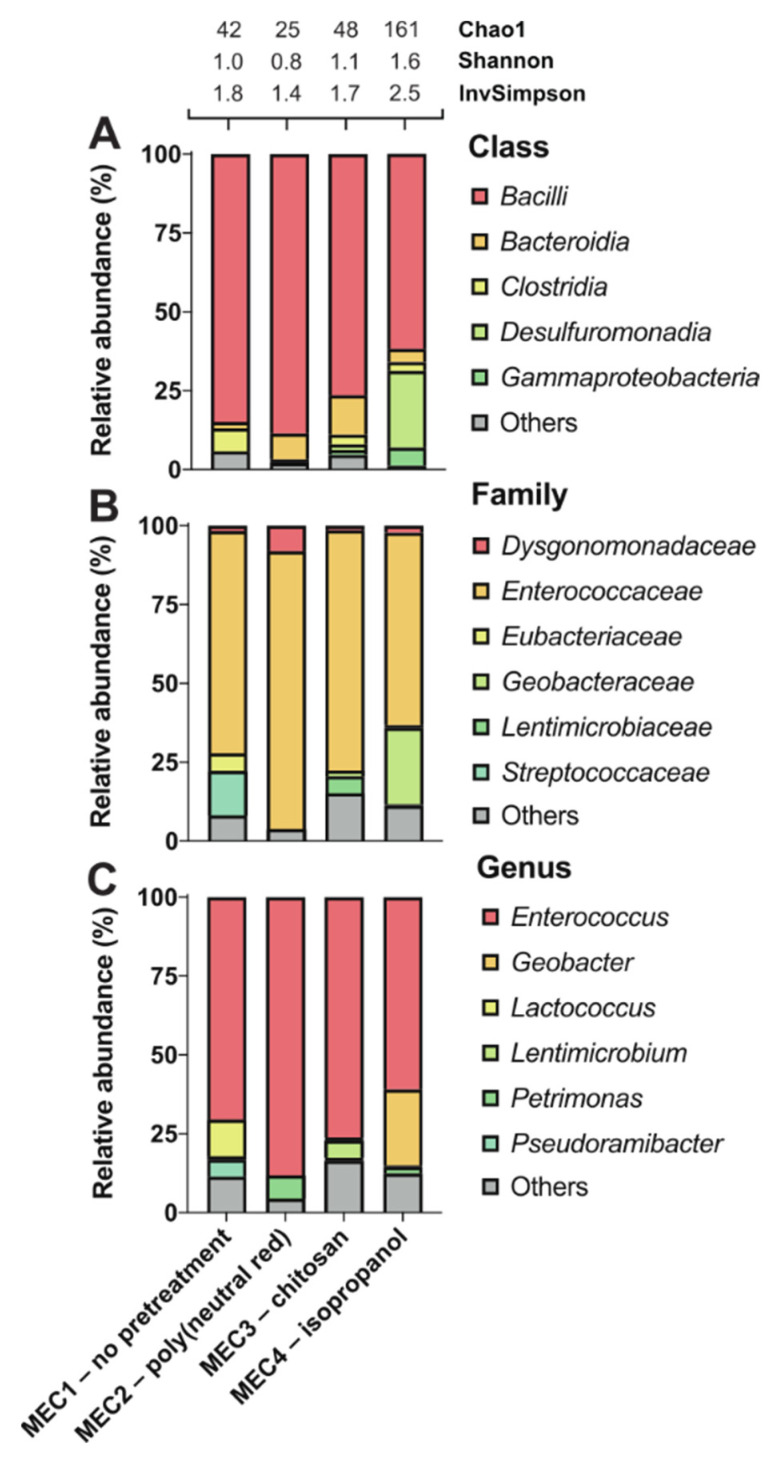
Effect of different electrode pretreatments on the enriched microbial communities of anode biofilm in MECs. Poly(neutral red) (MEC2), chitosan (MEC3), and isopropanol (MEC4) were used for the carbon felt electrode pretreatments, but the control electrode (MEC1) was not pretreated. Taxonomic profiles were set at the class (**A**), family (**B**), and genus (**C**) levels. Only representatives with a relative abundance >5% in at least one condition are shown. Detailed data are shown in Supplementary Table S3. Alpha diversity was estimated using the following indices: Chao1, Shannon, and Inverse Simpson.

The MECs performance may be affected by the biofilm, which comprises a syntrophic consortium of microorganisms adhered to the electrode. Therefore, the compositions of the microbial communities of biofilms developed on electrodes after different pretreatments were investigated (Figure 2).

Independent of pretreatments, the most abundant genus on all electrodes was Gram-positive *Enterococcus*. This genus was widely represented in all MECs: 71% (MEC1), 88% (MEC2), 76% (MEC3), and 61% (MEC4) (Figure 2). Although most studies have reported higher current production for Gram-negative bacteria, Gram-positive bacteria are also common members of microbial communities in BESs, as facultative anaerobic *Enterococcus faecalis* can perform extracellular electron transfer [47]. *Enterococcus* species belong to a heterogeneous group of lactic acid bacteria that ferment carbohydrates and produce lactic acid as a main fermentation product. This dominant presence of these species on all electrodes tested may be a consequence of the composition of the medium used in all MECs, which contained glucose as the main carbon source. In agreement with these results, strong biofilm formation by *Enterococcus faecalis* has been demonstrated in the presence of glucose [48]. Another Gram-positive lactic acid bacterium, *Lactococcus* (12%), was present on the untreated electrode (MEC1) and minimally present (<0.1%) on the pretreated electrodes (Figure 2). In a previous study, *Lactococcus lactis* was recognized for extracellular electron transfer by excretion of redox mediators [49]. Similarly, the existence of Gram-positive, strictly anaerobic *Pseudoramibacter* was detected (5%) on untreated but not found on pretreated electrodes. The co-occurrence of lactic acid bacteria and *Pseudoramibacter* have been observed in biogas plants [50]. *Pseudoramibacter* species are likely to metabolize lactate produced by lactic acid bacteria. On the other hand, Gram-negative fermentative bacterium *Petrimonas* (7%) co-occurred with dominant *Enterococcus* species at the poly(neutral red)-treated electrode in MEC2. Mesophilic anaerobe *Petrimonas sulfuriphila* isolated from a biodegraded oil reservoir [51] may degrade peptone and yeast extract to acetic acid and CO_2_ at the anode, as described previously [24]. Peptone and yeast extract were also added to the MECs growth medium, which affected enrichment of this species, together with poly(neutral red). Chitosan and its derivatives have been employed as effective agents to inhibit biofilm formation and attenuate virulence properties by various pathogenic bacteria [52]. However, the number of species detected in biofilm enriched after electrode pretreatment by chitosan was not significantly reduced (Figure 2). In addition to the dominant species *Enterococcus*, strictly anaerobic Gram-negative *Lentimicrobium* (6%) has been identified in the MEC3. Short rod-shaped *Lentimicrobium* has previously been recognized as the dominant genus in a bioelectrochemical reactor for nitrogen removal from wastewater [53]. Interestingly, chitosan increased the proportion of category “Others” (17%) (representing other microorganisms <5% abundance) at the genus level. This percentage was the highest share of “Others” compared to the rest of the electrode pretreatments and the control. One study reported the enrichment of *Proteobacteria*, *Firmicutes*, and *Bacteroidetes* as a dominant phyla on a composite chitosan-nitrogen-doped carbon nanotubes-polyaniline sponge anode, at which the addition of biocompatible chitosan seemed to increase the biodiversity of the biofilm, leading to enhanced current generation, due to synergistic effects of bacteria [54]. However, the number of species observed and diversity of MEC3 is similar to the untreated control and poly(neutral red)-treated anode biofilms (Figure 2).

In the isopropanol-treated electrode from MEC4, in addition to dominant *Enterococcus*, a considerable presence of *Geobacter* was found (24%), unlike other electrodes. *Geobacter* spp. belong to the most prominent exoelectrogenic bacteria representatives, which produce and transfer electrons to the anode with highly conductive pilis [55]. Bond et al. demonstrated that the nitrate-reducing species *Geobacter metallireducens* accepts electrons from an electrode [56]. Furthermore, *Geobacter sulfurreducens* produces high current densities at a moderate temperature [57]. The higher presence of *Geobacter* species positively affected the performance parameters (COD removal rate, COD removal efficiency, and Q rate) in MEC4, which were the highest compared to other MECs. This coincided with the previous study, reporting a linear current increase, with an increase of *G. sulfurredcuens* on the electrode surface [58]. As reported earlier [38], isopropanol pretreatment seems to reduce nitrogen functional groups on the electrode surfaces, especially the reduction of N-5 and N-6 groups with negatively charged nature. This leads to speculations that the reduction of negatively charged nitrogen groups improves the enrichment of negatively charged bacteria due to minor electrostatic repulsion [38]. However, other researchers claimed that a higher nitrogen content positively effects the bioanode performance, but enlightenment about the nitrogen types was not provided [46]. Moreover, the total oxygen content can be decreased by soaking with isopropanol and H_2_O_2_ [38], which may accelerate the attachment of electroactive microorganisms (such as *G. sulfurreducens)*. Furthermore, the treatment with H_2_O_2_ seems to improve the bacterial adhesion, because strong oxidation causes cracks and structural damage into carbon surfaces [38].

Interestingly, except for Gram-positive *Enterococcus*, most other genera identified on chitosan- (MEC3) and isopropanol-treated (MEC4) electrodes were Gram-negative bacteria. As mentioned before, Gram-negative bacteria have been noted for their high current production, which was observable in MEC3 and MEC4, producing higher electrical charges than MEC1 and MEC2. However, little is known about electroactivity in Gram-positive bacteria, which have a thicker peptidoglycan layer than Gram-negative bacteria but have no outer membrane [47]. The thicker cell wall seems to limit electron transfer directly from the cytoplasmic membrane, although there is no electron transfer requirement from the inner to outer membrane [59]. Our results showed that, not only the anode potential and the substrate composition influences the microbial consortium, as described in previous studies [60,61], but also the electrode pretreatment.

**Figure 3:**
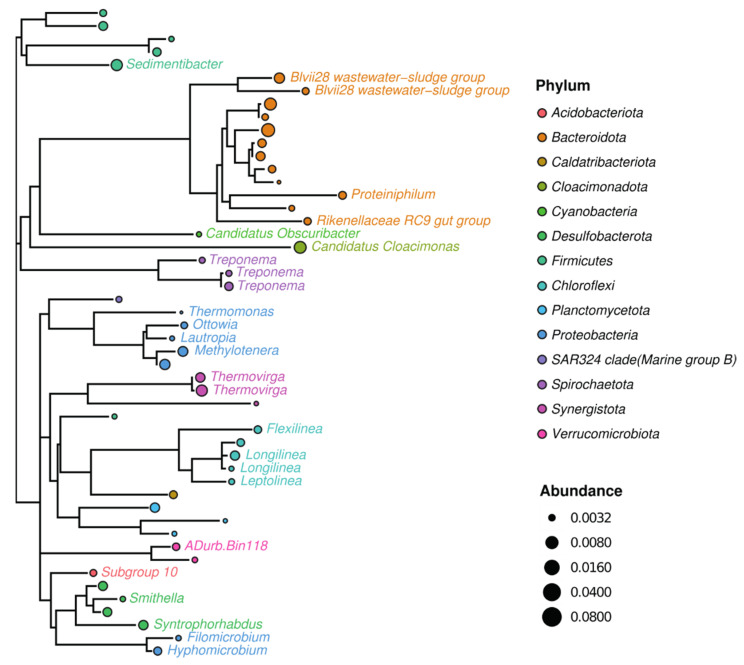
Phylogenetic tree, showing genetic relationships of sewage sludge community used for inoculation of all MECs. Only 50 most abundant representatives are shown. Circle color represents phylum taxa, and circle size corresponds to their relative abundance. The tree is labeled by color-coded representatives identified in the taxa of the genus.

**Figure 4:**
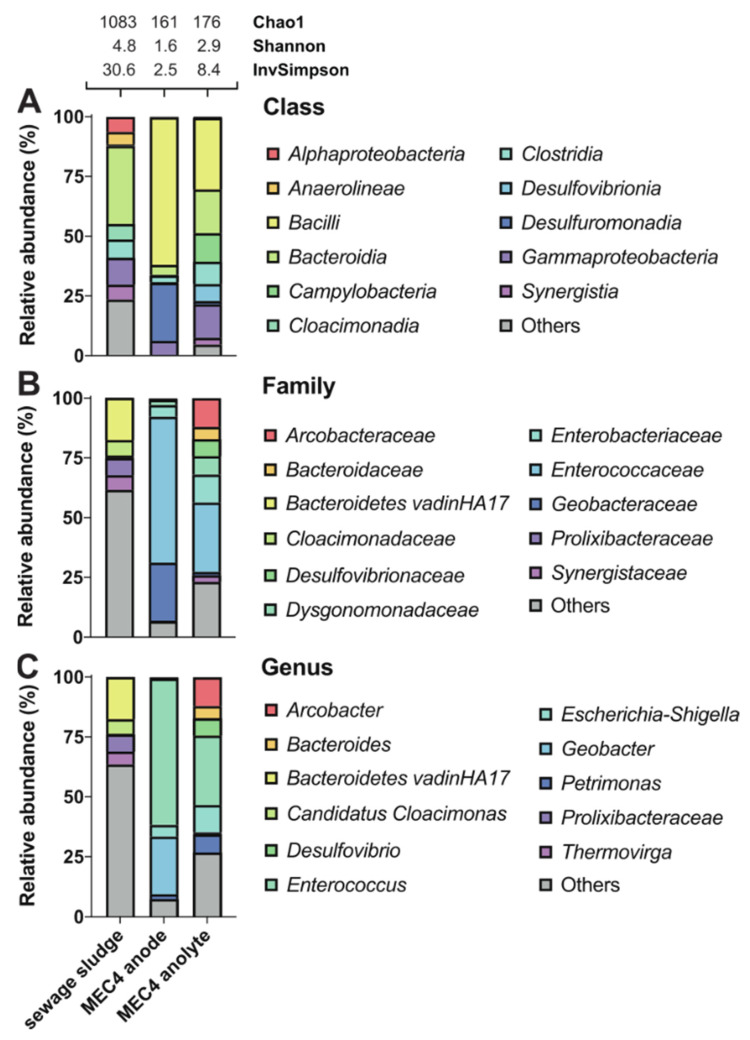
Effect of sewage sludge enrichment on the microbial community composition in MEC4. Sewage sludge represents the primary source for enrichment of electrochemically active microorganisms. MEC4 anode represents the microbial biofilm on the surface of the carbon felt electrode. MEC4 anolyte represents planktonic microorganisms in the anode chamber. Taxonomic profiles were set at class (**A**), family (**B**), and genus (**C**) levels. Only representatives with a relative abundance >5% in at least one condition are shown. Detailed data are shown in Supplementary Table S4. Alpha diversity was estimated using the following indices: Chao1, Shannon, and Inverse Simpson.

The sewage sludge community from the wastewater treatment plant used to enrich electroactive microorganisms in all MECs was analyzed by culture-independent metagenomic sequencing. The 50 most abundant representatives were phylogenetically divided into 14 different phyla, of which 21 were known genera (Figure 3). The microbiome of sewage sludge contained 1083 observed species and was slightly dominated by *Bacteroidetes* environmental groups, such as Vadin HA17 (18%) (Figure 4), also found in anaerobic digesters [62]. Only three other genera were identified above 5% of the total representatives, such as *Candidatus Cloacimonas* (6%), *Prolixibacteraceae* (7%), and *Thermovirga* (5%) (Figure 4). *Candidatus Cloacimonas* is probably a syntrophic bacterium present in many anaerobic digesters [63]. *Thermovirga* is a Gram-negative, anaerobic, and moderately thermophilic genus of bacteria isolated from oilfield environments [64]. These results highlight bacterial clades whose members are involved in anaerobic digestion, which involves the fermentation of amino acids when deriving most of their carbon and energy.

Furthermore, the metagenomic analysis was used to determine which bacteria were enriched from sewage sludge inoculum in the BES. As the best performance was achieved using the electrode with isopropanol pretreatment (MEC4), biofilm from the anode and planktonic microorganisms from the anolyte were selected for the metagenomic analysis (Figure 4). By correlating all alpha diversity indices, the sewage sludge community showed the highest degree of diversity, corresponding to many species with well-balanced abundances. The richness of sewage sludge consortium was also evident in high representation of taxa below 5% (Others) at genus (total of 64%) and family (total of 62%) levels. Not until the class level did the share of Others fall to 24% (Figure 4).

After BES enrichment using sewage sludge inoculum, the diversity of the biofilm attached to the anode and diversity of the planktonic community in the anolyte decreased significantly (Figure 4). Still, the microbial diversity was higher in the planktonic community when compared to the biofilm. The biofilm from MEC4 anode contained two dominant genera, namely *Enterococcus* and *Geobacter*, as mentioned above. However, these genera were not detected (or were only in small amounts) in the sewage sludge, indicating strong selection during enrichment in all MECs. Growth of *Enterococcus* was most likely favored by the culture conditions, while pretreatment of the electrode with isopropanol potentially enhanced growth of *Geobacter*. *Geobacter* is widespread in environmental samples, especially in soil. In a previous paper, *Geobacter* was the dominant species on all electrodes independent from the used inoculum, which leads to the assumption that *Geobacter* grows better at MFC conditions than any other bacteria [65]. Furthermore, this group also observed small differences regarding the dominant microbial communities at COO^−^ and SO_2_NH_2_ pretreated anodes. Others hypothesized that *Geobacter* prefers adhesion on hydrophilic and positively charged electrode surfaces [66]. If *Geobacter* is a dominant species on the electrode, as in MEC4, assisted due to electrochemical culture conditions and appropriate electrode pretreament, this may supress other genera, such as *Enterococcus*. Further, cultivation of *Geobacter* at electrochemical conditions improves the formation of biofilms and production of extracellular polymeric substances, mainly composed of polysaccharides and proteins, such as cytochrome, which is probably involved in extracellular electron transfer [67,68]. In addition, the *Enterobacteriaceae* family was detected at the MEC4 anode with a relative abundance of almost 5% (Figure 4), of which *Enterobacter cloacae* have been reported for power production [69].

On the other hand, the planktonic community from MEC4 anolyte was represented by *Enterococcus* (29%), *Arcobacter* (12%), *Escherichia-Shigella* (11%), *Petrimonas* (8%), *Desulfovibrio* (7%), and *Bacteroides* (5%) (Figure 4). *Arcobacter butzleri* is the first identified exoelectrogenic *Epsilonbacteria* so far [70]. However, direct electrode contacts or short distances between microbes and electrodes are required for extracellular electron transfer and subsequent current production [55]. Other authors also speculated that *Arcobacter spp*. are located in BESs because of their preference for microaerophilic conditions [71]. *Escherichia* spp. are well-known for electron production and extracellular electron transfer [72]. Furthermore, *Escherichia* spp. might cooperate with other microorganisms, and resulting synergies can increase power production. For instance, *E. coli* can cooperate with anaerobic *G. sulfurreducens* by removing oxygen from the surrounding and providing anaerobic conditions [73]. The electroactive representatives of *Desulfovibrio* spp. are sulfate-reducing *Desulfovibrio desulfuricans* and hydrogen-producing *Desulfovibrio paquesii* [74].

## 4. Conclusions

This study investigated the effects of various electrode pretreatments with poly(neutral red), chitosan, and isopropanol on anodic biofilm community composition. Furthermore, the microbial enrichment on the electrode and the anolyte was examined and compared with the sewage sludge inoculum’s microbial composition. Four MECs consisting of organic-oxidizing bioanodes and methane-producing biocathodes were operated by applying a potential of 400 mV vs. Ag/AgCl (3M NaCl) on the bioanode. Different pretreatment methods for carbon felt electrodes were tested, and each MEC was equipped with one pretreatment method. After 1 month of operation, the metagenomic analysis revealed that the carbon felt electrode pretreatment offers specific culture conditions, enabling the enrichment of specific genera, whereby the bacterial diversity was reduced. Although the main dominant genus on all electrodes was *Enterococcus*, certain bacteria were only identified depending on the electrode modifications. Moreover, the bacterial diversity of the biofilm was found to be lower when compared to the anolyte sample. Further research is necessary to examine electrochemically-active microbial biofilms, enriched on various bioelectrodes more profoundly, and investigate the relation and cooperation of those with planktonic communities to enhance performance of BESs and related applications, such as the MFC-based biosensor.

## Figures and Tables

**Table 1:** Comparison of following monitored parameters in MEC1–4: COD removal rate, COD removal efficiency, CE anode, Q rate, CH_4_ production rate, and CE cathode.

Cell Name	COD Removal Rate [mg/L/d]	COD Removal Efficiency [%]	CE Anode [%]	Q Rate [C/d]	CH_4_ Production Rate [mmol/L/d]	CE Cathode [%]	Ref.
MEC1	40	25	64	60	0.11	39	[40]
MEC2	55	52	75	110	0.41	66	[40]
MEC3	65	56	76	130	0.41	57	[40]
MEC4	69	76	75	137	0.44	58	This work

## Data Availability

Datasets generated and analyzed during the current study are available in the NCBI Sequence Read Archive under project number BioProject ID: PRJNA718863.

## Supplementary Materials

The following are available online at https://www.mdpi.com/article/10.3390/bios11060170/s1, Table S1: The list of primers used in 16S rRNA gene amplicon sequencing analysis; Table S2: Amplicon sequence variants table of all bacteria detected; Table S3: Supplementary data to Figure 2; Table S4: Supplementary data to Figure 4.
